# Effect of Sleep Characteristics on Rapid Estimated GFR Decline in Adults with Normal Kidney Function

**DOI:** 10.1016/j.ekir.2025.03.042

**Published:** 2025-04-03

**Authors:** Mohammadreza Akbari, Seyed Alireza Mirhosseini, Kimia Falamarzi, Hossein Molavi Vardanjani, Zahra Rahimian, Azizallah Dehghan, Hiva Alipanah, Jamshid Roozbeh

**Affiliations:** 1 Nephro-Urology Research Center, Shiraz University of Medical Sciences, Shiraz, Iran; 2 MD-MPH Department, School of Medicine, Shiraz University of Medical Sciences, Shiraz, Iran; 3 Cardiovascular Research Center, School of Medicine, Shiraz University of Medical Sciences, Shiraz, Iran; 4 Student Research Committee, Shiraz University of Medical Sciences, Shiraz, Iran; 5 MD-MPH Program, School of Medicine, Research Center for Traditional Medicine and History of Medicine, Shiraz University of Medical Sciences, Shiraz, Iran; 6 Noncommunicable Diseases Research Center, Fasa University of Medical Sciences, Fasa, Iran; 7 Department of Physiology, School of Medicine, Fasa University of Medical Sciences, Fasa, Iran

**Keywords:** cohort study, daytime nap, renal function decline, sleep duration, sleep quality

## Abstract

**Introduction:**

This study was conducted to evaluate the association between self-reported sleep characteristics and rapid decline of estimated glomerular filtration rate (eGFR) in adults with initially normal kidney function.

**Methods:**

Data from participants in the reevaluation phase of the Fasa Adult Cohort Study (FACS) were analyzed. Rapid eGFR decline was defined as an annual decrease > 3 ml/min per 1.73 m^2^ and rapid eGFR percent decline was defined as an annual decrease > 10th percentile of study sample. Daily sleep duration, daytime napping, sleep onset latency (SOL), the use of sleeping pills and daytime sleepiness were assessed at baseline through interviews conducted by trained nurses. Poisson regression models were applied to evaluate the risk of outcomes across sleep characteristics.

**Results:**

A total of 2810 participants, of whom 53.7% were female, with a mean age of 49.8 (± 8.3) yrs and a baseline eGFR of 88.5 (± 10.6) were studied. Regular daytime napping was associated with a reduced risk of rapid eGFR decline in individuals aged > 50 yrs (adjusted relative risk [RR]: 0.64, 95% confidence interval [CI]: 0.48–0.86). Specifically, naps exceeding 90 minutes daily were associated with a 49% reduced risk compared with nonnappers (adjusted RR: 0.51, 95% CI: 0.33–0.81). Among participants aged < 50 yrs, sleeping < 7 h/d was linked to a higher likelihood of rapid eGFR percent decline (adjusted RR:1.78, 95% CI: 1.12–2.82).

**Conclusion:**

Regular daytime napping, especially in older adults, may be protective against kidney function decline, whereas short sleep duration may accelerate eGFR decline in younger adults. Addressing sleep habits may aid in preventing chronic kidney disease (CKD).

## Graphical abstract

**Figure:**
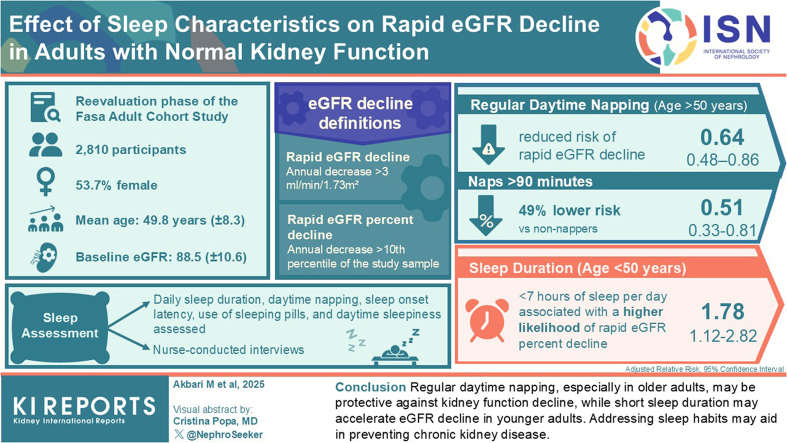

See Commentary on Page 1613

CKD, which affects about 10% of the world's population, is characterized by the gradual loss of kidney function.^1^ With its increasing prevalence, high treatment costs, and poor outcomes, it has become a major public health concern worldwide.^2^ Because early kidney function decline is often asymptomatic, identifying modifiable risk factors is crucial for CKD prevention.^3^

Sleep plays a critical role in overall health, and disturbances in sleep duration, quality, and patterns have been linked to cardiometabolic conditions, including cardiovascular disease, obesity, and diabetes.^4^, ^5^, ^6^ Sleep is a complex behavior, and different aspects may influence renal function through distinct mechanisms. Short sleep duration is associated with sympathetic nervous system activation,^7^ which may contribute to glomerular hypertension and kidney damage; whereas daytime napping may serve as a compensatory mechanism for insufficient nighttime sleep. Prolonged SOL has been linked to biological aging and may reflect underlying changes in glomerular health.^8^ Sleeping pill use and daytime sleepiness often reflect poor sleep quality, which may contribute to cardiovascular dysregulation.^9^

Few cohort studies suggest that poor sleep quality and inadequate duration of sleep might negatively affect kidney function.^10^, ^11^, ^12^ However, these results are not consistent, because some studies link both short and long sleep durations to a higher risk of CKD, whereas others find an association only with short sleep or primarily with proteinuria rather than CKD development.^13^, ^14^, ^15^ These inconsistencies in findings highlight the need for further investigations to determine the precise role of sleep in the development of CKD and as a modifiable risk factor for CKD prevention.

We conducted this analysis using data from the baseline and reevaluation phase of the FACS study to assess the association between multiple sleep characteristics, including daily sleep duration, daytime napping, prolonged SOL, sleeping pill use, and daytime sleepiness; and rapid eGFR decline in individuals with initially normal kidney function. Unlike most previous research, we simultaneously examined multiple sleep parameters within the same model. This approach allowed us to differentiate the independent effect of each sleep characteristic on rapid renal function decline, providing a clearer understanding of their distinct contributions.

## Methods

### Study Population

This study is a secondary analysis of baseline and reevaluation data of the FACS study. Between October 2014 and September 2016, individuals aged between 35 and 70 yrs who lived in Sheshdeh and Qarabolagh districts in southern Iran were enrolled into FACS, a cohort aimed at evaluating noncommunicable diseases in a general Iranian population.^16^ After obtaining informed written consent from each participant, comprehensive questionnaires, measurements, physical examinations, and sampling were conducted. This study was approved by the ethics committee of Shiraz University of Medical Sciences under reference number IR.SUMS.MED.REC.1403.536.

The FACS conducted annual telephone follow-ups to monitor the incidence of various noncommunicable diseases. Since September 2021, the reevaluation phase with the same protocol as the first phase has been conducted for 3029 of the participants of the first phase selected using a random sampling method.^16^ For this study, baseline data on sleep characteristics and covariates were collected from 2014 to 2016, and eGFR decline was calculated using eGFR measurements of baseline and reevaluation phase in 2021.

Participants included in this analysis were those who entered the reevaluation phase in 2021. Exclusions were applied based on baseline data, removing individuals who had an eGFR < 60 ml/min per 1.73 m^2^, proteinuria detected as +1 or more on a dipstick, a previous physician-diagnosed CKD, or missing data on all sleep characteristics.

### Exposure Variables

Sleep characteristics were assessed at baseline through interviews conducted by trained nurses. To evaluate daily sleep duration, participants were asked the following 2 questions: “What is your average sleep duration at night?” and “In 24 hours, how much do you sleep in the evening or midday?” Daily sleep duration was calculated by summing the responses and categorized into 3 groups: < 7 h, 7 to 9 h, and ≥ 9 h. For daytime napping, participants were also asked: “Do you nap during the day on 3 or more days per week?” Those who answered "No" were classified as nonnappers. Among regular nappers, nap duration was further categorized into ≤ 30 min, 30 to 90 min, and > 90 min in 24 h.

SOL was assessed by asking, “How long does it take, in minutes, from the time you go to bed to fall asleep?” A habitual SOL of more than 60 minutes was considered prolonged, because SOL > 1 h has been associated with all-cause mortality.^17^

In addition, we evaluated the use of sleeping pills and daytime sleepiness. Sleeping pill use was assessed with the question: “Do you take sleeping pills more than 2 days a week?” Daytime sleepiness was measured by asking: “Do you doze off involuntarily during the day when you are not engaged in activity?”

### Outcome Variable

eGFR was calculated using the CKD-Epidemiology Collaboration formula.^18^ The CKD-Epidemiology Collaboration equation is recognized as superior to the Modification of Diet in Renal Disease equation for glomerular filtration rate estimation, particularly in individuals with eGFR values > 60 ml/min per 1.73 m^2^, which applies to the participants in this study.^19^ Because the CKD-Epidemiology Collaboration equation was developed using IDMS-traceable creatinine assays, we reduced our serum creatinine values by 5% before calculating eGFR to ensure compatibility with the CKD-Epidemiology Collaboration equation, following guidance from the previous literature.^20^^,^^21^

Annual eGFR change rate was calculated as: (baseline eGFR − reevaluation eGFR) ÷ duration between 2 laboratory tests in years. The primary end point, rapid eGFR decline, is defined as an annual eGFR change rate > 3 ml/min per 1.73 m^2^/yr. We also calculated the annual eGFR percent change rate as: (annual eGFR change rate ÷ baseline eGFR) × 100. For the definition of rapid decline percent, our secondary end point, we used the 10th percentile of annual eGFR percent change (annual eGFR decline > 3.76%). Both outcomes were binary variables, indicating the presence or absence of rapid decline based on these thresholds.

### Covariates

Several factors can confound the relationship between sleep characteristics and renal function, so various potential confounders were evaluated in this study. These variables, including age, sex, ethnicity, smoking status, and body mass index, were all obtained through interviews or physical examinations following standard protocols at baseline.

Participants were divided into tertiles of socioeconomic status based on a wealth score index, which was estimated using multiple correspondence analysis of participants' assets. This index reflects the overall wealth status of individuals or households based on their access to various assets.^22^ Participants were categorized into 3 groups based on their educational level as follows: (i) < 5 yrs of education, (ii) 5 to 7 yrs of education, and (iii) at least 8 yrs of education (secondary degree). Physical activity is reported using the Metabolic Equivalent of Task score, calculated over 24 hours,^23^ and divided into 3 tertiles. A history of cardiovascular disease was recorded for those with a previous diagnosis of acute or chronic coronary syndrome, stroke (cerebrovascular accident), or peripheral artery disease. Blood pressure was measured twice on both the left and right arms while the participant was calm and seated. The final blood pressure reading was the average of the second measurement from each arm. Uncontrolled hypertension was defined as systolic blood pressure > 140 mm Hg or diastolic blood pressure > 90 mm Hg. The use of nonsteroidal antiinflammatory drugs or angiotensin-converting enzyme inhibitors or angiotensin receptor blockers was defined as regular use within the past 3 months before the interview. Dyslipidemia was defined as having total cholesterol > 220 mg/dl, high-density lipoprotein < 35 mg/dl, low-density lipoprotein > 160 mg/dl, or triglycerides > 200 mg/dl.

### Statistical Analysis

Descriptive statistics were presented as mean (± SD) or median (interquartile range) for continuous variables, and frequency (percentage) for categorical variables. Poisson regression analysis was employed to build models analyzing the relationship between the incidence of outcomes and sleep characteristics, estimating the RR and 95% CI. Adjusted models include sleep variables and age, sex, ethnicity (Fars, not Fars), baseline eGFR, uncontrolled hypertension, diabetes mellitus, cardiovascular disease (yes, no), body mass index, socioeconomic status (tertiles), education level (categorical), physical activity (tertiles), smoking (yes, no), dyslipidemia (yes, no), and using nonsteroidal antiinflammatory drug or angiotensin-converting enzyme inhibitors or angiotensin receptor blockers. Because a significant interaction between baseline age and daytime napping in the adjusted models, we stratified the analysis by age groups, with participants aged < 50 yrs and those aged > 50 yrs. For daytime napping, we replaced the binary variable (regular daytime nap) with nap duration in the models to assess the dose-response relationship. All statistical analyses were conducted using R software (V 4.2.1; R Core Team 2022), with a *P*-value < 0.05 considered statistically significant.

## Results

**Figure 1:**
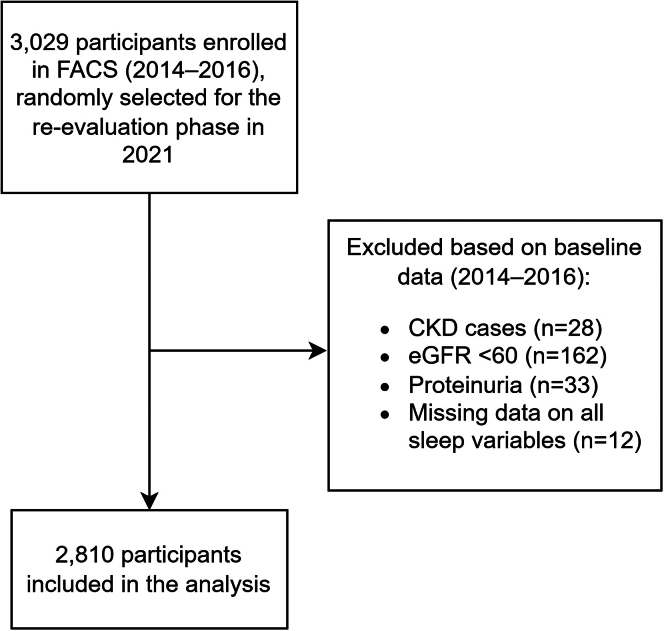
The flowchart of the study design. CKD, chronic kidney disease; eGFR, estimated glomerular filtration rate; FACS, Fasa Adult Cohort Study.

According to the provided flowchart, of the 3029 participants in the reevaluation phase of FACS, 2810 individuals were included (Figure 1). Of the 3029 participants in the FACS study who had provided 2 blood samples, 2810 individuals were included. Among them, 1400 were aged < 50 yrs, and 1410 aged > 50 yrs. The sample included 1510 females (53.7%). These participants had a mean age of 49.8 (± 8.3) yrs and baseline eGFR of 88.5 (± 10.6). The interval between the time of collection of the 2 blood samples was 76.7 (72.3–81.8) mos. The baseline characteristics of the study population are summarized in Table 1. The characteristics of participants across sleep variables are presented in Supplementary Tables S1 and S2.

**Table 1:** Baseline characteristics of study participants

Variable	Overall	Baseline age < 50 yrs	Baseline age ≥ 50 yrs	Count of missing data
Age (yrs)	50 [44,56]	44 [39,47]	56 [53,60]	0
eGFR (ml/min per 1.73 m^2^)	88.1 (80.3–96.6)	89.8 [84.4–100.6]	83.8 [78.7–93.3]	0
BMI (kg/m^2^)	25.7 [22.6–28.7]	26 [22.8–29.2]	25.3 [22.3–28.2]	4
Female	1510 (54)	883 (63)	627 (44)	0
Socioeconomic status				0
Low	937 (33)	436 (31)	501 (36)	
Moderate	937 (33)	459 (33)	478 (34)	
High	936 (33)	505 (36)	431 (31)	
Fars ethnicity	1496 (53)	752 (54)	744 (53)	0
Education years				0
Low	1372 (49)	480 (34)	892 (63)	
Moderate	989 (35)	624 (45)	365 (26)	
High	449 (16)	296 (21)	153 (11)	
Currently smoker	473 (17)	208 (15)	265 (19)	0
Uncontrolled HTN	453 (16)	158 (11)	295 (21)	0
Diabetes	369 (13)	112 (8)	257 (18)	1
CVD	306 (11)	73 (5)	233 (17)	1
NSAID use	137 (5)	43 (3)	94 (7)	0
ACEi/ARB use	192 (7)	45 (3)	147 (10)	0
Physical activity				0
Low	942 (34)	488 (35)	454 (32)	
Moderate	936 (33)	466 (33)	470 (33)	
High	932 (33)	446 (32)	486 (34)	
Dyslipidemia	845 (30)	377 (27)	468 (33)	1
Daytime nap				0
Nonnapper	1126 (40)	488 (35)	638 (45)	
nap for ≤ 30 min	226 (8)	108 (8)	118 (8)	
nap for 30–90 min	860 (30)	461 (33)	399 (28)	
nap for > 90 min	598 (21)	343 (24)	255 (18)	
Sleep duration				0
< 7 h	725 (26)	275 (20)	450 (32)	
7–9 h	1307 (47)	663 (47)	644 (46)	
≥ 9 h	778 (28)	462 (33)	316 (22)	
Prolonged sleep latency	189 (7)	70 (5)	119 (9)	98
Sleeping pills use	200 (7)	96 (7)	104 (7)	0
Daytime sleepiness	899 (32)	390 (28)	509 (36)	0

ACEi, angiotensin-converting enzyme inhibitor; ARB, angiotensin II receptor blocker; BMI, body mass index; CVD, cardiovascular disease; eGFR, estimated glomerular filtration rate; HTN, hypertension; NSAID, nonsteroidal anti-inflammatory drug.

Variables are presented as mean ± SD, median [interquartile range] or numbers (percentages).

A total of 398 individuals (14.2%) experienced a rapid eGFR decline, and 281 (10.0%) experienced rapid eGFR percent decline. The regression analysis revealed that among the evaluated sleep variables, only daytime napping was significantly associated with rapid eGFR decline (Supplementary Table S3). In participants aged > 50 yrs, regular daytime napping remained a protective factor against rapid eGFR decline (adjusted RR: 0.64, 95% CI: 0.48–0.86). The crude and adjusted RRs for sleep variables are provided in Table 2. Similar results were observed when analyzing the rapid percent decline in eGFR; however, in this case, both daytime napping and < 7 hours of sleep were significant factors (Table 2).

**Table 2:** Crude and adjusted relative risk (RR) of models developed for the relationship between sleep profile variables and rapid eGFR decline or rapid eGFR percent decline among those aged < 50 years and those > 50 years

Sleep profile variables	Baseline age < 50 yrs				Baseline age ≥ 50 yrs			
	Crude model		Adjusted model^a^ (*N* = 1353)		Crude model			Adjusted model^a^ (*N* = 1353)
	RR (95% CI)	*P*-value	RR (95% CI)	*P*-value	RR (95% CI)	P-value	RR (95% CI)	*P*-value
Rapid eGFR decline								
Daily sleep duration (ref: 7–9 h)								
< 7 h	1.33 (0.94–1.86)	0.104	1.38 (0.97–1.98)	0.075	0.97 (0.72–1.30)	0.832	0.91 (0.68–1.23)	0.556
> 9 h	1.22 (0.90–1.65)	0.195	1.27 (0.92–1.76)	0.146	0.96 (0.69–1.33)	0.784	1.20 (0.84–1.72)	0.305
Regular napping	1.02 (0.77–1.35)	0.880	1.03 (0.74–1.42)	0.872	0.64 (0.50–0.83)	0.001	0.64 (0.48–0.86)	0.003
Prolonged sleep onset latency	1.69 (1.05–2.71)	0.029	1.58 (0.98–2.56)	0.063	1 (0.63–1.59)	0.984	1.06 (0.66–1.71)	0.811
Sleeping pill use	0.99 (0.58–1.67)	0.959	0.92 (0.52–1.62)	0.766	0.78 (0.45–1.34)	0.366	0.66 (0.36–1.21)	0.175
Daytime sleepiness	0.99 (0.74–1.33)	0.933	1 (0.74–1.36)	0.994	0.91 (0.70–1.19)	0.494	0.83 (0.63–1.10)	0.195
Rapid eGFR percent decline								
Daily sleep duration (ref: 7–9 h)								
< 7 h	1.64 (1.07–2.51)	0.023	1.78 (1.12–2.82)	0.015	0.98 (0.70–1.36)	0.901	0.83 (0.59–1.17)	0.279
> 9 h	1.25 (0.84–1.87)	0.273	1.46 (0.94–2.27)	0.091	0.88 (0.60–1.30)	0.534	1.08 (0.72–1.62)	0.720
Regular napping	1.15 (0.80–1.67)	0.444	1.11 (0.72–1.72)	0.630	0.64 (0.47–0.85)	0.002	0.66 (0.47–0.91)	0.012
Prolonged sleep onset latency	1.96 (1.11–3.48)	0.021	1.58 (0.86–2.90)	0.139	1.13 (0.69–1.86)	0.629	1.03 (0.62–1.70)	0.916
Sleeping pill use	1.23 (0.67–2.28)	0.500	1.04 (0.53–2.05)	0.913	0.66 (0.33–1.30)	0.227	0.57 (0.29–1.12)	0.103
Daytime sleepiness	0.9 (0.61–1.33)	0.606	0.89 (0.59–1.35)	0.591	1.02 (0.76–1.38)	0.878	0.91 (0.67–1.25)	0.571

ACEi, angiotensin-converting enzyme inhibitor; ARB, angiotensin II receptor blocker; BMI, body mass index; CI, confidence interval; eGFR, estimated glomerular filtration rate; HTN, hypertension; NSAID, nonsteroidal antiinflammatory drug; ref, reference; RR, relative risk.

a Adjusted models include all sleep variables and are adjusted for age, sex, ethnicity [Fars, not Fars], baseline eGFR, uncontrolled HTN, diabetes mellitus, history of cardiovascular disease [yes, no], BMI, socioeconomic status [tertiles], education level [categorical], physical activity [tertiles], smoker [yes, no], dyslipidemia [yes, no], and using NSAID or ACEi/ARB.

**Figure 2:**
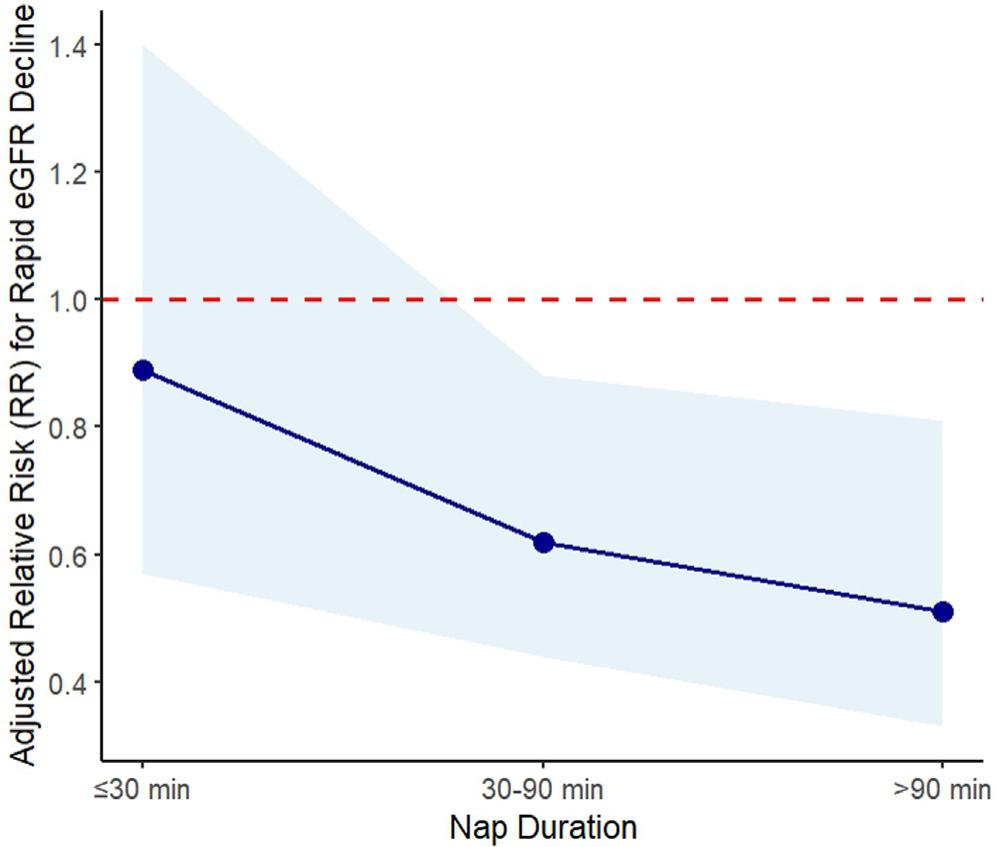
Dose-response relationship between nap duration and the risk of rapid eGFR decline in those aged > 50 years. The figure illustrates the association between nap duration and the adjusted relative risk of rapid eGFR decline. The solid line represents the estimated relative risk, whereas the shaded area indicates the 95% confidence interval. A reference line at relative risk = 1 shows no effect. eGFR, estimated glomerular filtration rate.

By replacing the binary variable of regular daytime nap with nap duration, a dose-response protective effect was observed in participants aged > 50 yrs. After adjusting for confounders, those who regularly napped for > 90 minutes were 49% less likely to experience rapid eGFR decline compared with nonnappers (Table 3, Fig 2).

**Table 3:** Crude and adjusted relative risk of analysis of dose response relationship between daytime napping and rapid eGFR decline and rapid eGFR percent decline among those aged < 50 years and those aged > 50 years

Daytime napping (ref: nonnapper)	Baseline age < 50 yrs				Baseline age ≥ 50 yrs			
	Crude model		Adjusted model^a^ (*N* = 1353)		Crude model		Adjusted model^a^ (*N* = 1353)	
	RR (95% CI)	*P*-value	RR (95% CI)	*P*-value	RR (95% CI)	*P*-value	RR (95% CI)	*P*-value
Rapid eGFR decline								
nap for ≤ 30 min	0.89 (0.51–1.55)	0.682	0.96 (0.53–1.74)	0.892	0.79 (0.49–1.27)	0.332	0.89 (0.57–1.40)	0.629
nap for 30–90 min	0.91 (0.66–1.27)	0.595	0.99 (0.69–1.41)	0.941	0.65 (0.47–0.89)	0.022	0.62 (0.44–0.88)	0.007
nap for > 90 min	1.21 (0.87–1.68)	0.261	1.13 (0.75–1.68)	0.561	0.56 (0.37–0.84)	0.001	0.51 (0.33–0.81)	0.004
Rapid eGFR percent decline								
nap for ≤ 30 min	1.07 (0.53–2.15)	0.849	1.12 (0.57–2.20)	0.738	0.65 (0.36–1.18)	0.161	0.70 (0.39–1.26)	0.236
nap for 30–90 min	1.09 (0.71–1.67)	0.705	1.09 (0.67–1.77)	0.728	0.70 (0.50–1.00)	0.048	0.70 (0.47–1.03)	0.075
nap for > 90 min	1.28 (0.82–1.98)	0.284	1.15 (0.66–2.02)	0.626	0.52 (0.33–0.84)	0.007	0.55 (0.33–0.90)	0.018

ACEi, angiotensin-converting enzyme inhibitor; ARB, angiotensin II receptor blocker; BMI, body mass index; CI, confidence interval; eGFR, estimated glomerular filtration rate; HTN, hypertension; NSAID, nonsteroidal antiinflammatory drug; ref, reference; RR, relative risk.

a Adjusted models include all sleep variables and are adjusted for age, sex, ethnicity [Fars, not Fars], baseline eGFR, uncontrolled HTN, diabetes mellitus, history of cardiovascular disease [yes, no], BMI, socioeconomic status [tertiles], education level [categorical], physical activity [tertiles], smoker [yes, no], dyslipidemia [yes, no] , and using NSAID or ACEi/ARB.

## Discussion

In this study, we observed that in adults aged ≥50 yrs, regular daytime napping was associated with a significantly lower risk of rapid renal function decline. This association was dose-dependent, with naps lasting > 1.5 h/d associated with a lower risk of rapid eGFR decline by half. Interestingly, among participants aged < 50 yrs, those who slept for < 7 h/d had a higher likelihood of experiencing rapid eGFR percent decline. However, no significant associations were observed between SOL, daytime sleepiness, or the use of sleep medications and rapid renal function decline.

### Sleep Duration

Our results suggest that shorter sleep duration increases the risk of rapid eGFR percent decline in younger adults, although no statistically significant association was observed in older adults or for rapid eGFR decline in either age group. Findings from previous research on the longitudinal effect of sleep duration on kidney function have been inconsistent. Some studies found no association between sleep duration and kidney function,^12^^,^^13^ whereas others reported that both short and long sleep durations are harmful to kidney health^24^^,^^25^ and some identified only short sleep duration as a risk factor.^26^^,^^27^

A key limitation of most previous studies is their focus on only 1 or 2 sleep characteristics, whereas our study provides a more comprehensive perspective by assessing multiple sleep dimensions, including SOL, sleeping pill use, daytime sleepiness, and daytime napping, in addition to daily sleep duration. By adjusting for all these sleep characteristics simultaneously, our study offers a more precise estimate of each factor’s independent effect. Another major source of variability among studies is the different cutoffs used to define normal and abnormal sleep duration, as well as differences in how sleep duration was measured; some studies considered only nighttime sleep, whereas others included total 24-h sleep.

Short sleep duration may activate the sympathetic nervous system and increased sympathetic nervous system activity might lead to renal vasoconstriction and glomerular hypertension which ultimately affects kidney function and contributes to glomerular filtration rate decline and CKD development.^25^^,^^28^, ^29^, ^30^ Moreover, sleep deprivation increases proinflammatory cytokines and results in glomerular endothelial dysfunction and renal damage.^24^^,^^31^

According to our findings, individuals aged < 50 yrs who slept < 7 h/d were more likely to experience a rapid eGFR percent decline compared with their older counterparts. This difference may stem from the fact that sleep duration decreases with age and healthy elderly individuals are more tolerant of sleep deprivation than younger individuals.^32^, ^33^, ^34^ Regarding the physiological mechanisms, it can be explained that as people age, the number of ventrolateral preoptic nuclei of the hypothalamus decreases, leading to reduced sleep duration.^34^ This suggests that the impact of sleep duration on the risk of developing incident chronic disease conditions should be evaluated separately in the middle-aged and elderly populations because of their physiological differences. In this regard, a study indicated that the risk thresholds for new-onset chronic disease conditions were 7 hours of sleep in middle-aged population and 6 hours in the elderly.^35^

### Daytime Napping

To the best of our knowledge, only 3 longitudinal studies have investigated the effect of daytime napping on renal function, and the results have been contradictory. One study found a protective effect of daytime napping on new-onset nephropathy among older Chinese adults, but not in middle-aged individuals, and reported a negative linear relationship between longer nap durations and the risk of kidney disease in the elderly.^35^ This finding is generally consistent with ours, because we also demonstrated that napping is protective in older adults and observed a dose-response relationship. A prospective cohort study among middle-to-older-aged British population with a follow-up period of 11 yrs indicated that daytime napping was identified as a risk factor for incident CKD.^11^ Another longitudinal study in China found no statistical association between daytime napping and increased risk of CKD.^12^ Further studies are needed to explore the reasons for variation across these findings, with lifestyle factors likely playing important roles.

Jiang *et al.* revealed that daytime napping might diminish the adverse effects of short nighttime sleep duration and compensate for the lack of nocturnal sleep duration.^36^ A previous study found that daytime napping reduced urinary catecholamine levels, which were increased after sleep restriction and may cause renal vasoconstriction and damage, suggesting that regular napping might attenuate the risk for renal damage caused by sympathetic activation.^36^^,^^37^ As mentioned above, studies which assess the effects of daytime napping on kidney function are scarce and the results are inconsistent. Therefore, further work is required to address this issue and explain the exact mechanisms that may link daytime napping to kidney function and CKD incident.

### SOL, Sleeping Pill Use, and Daytime Sleepiness

In this study, no significant associations were observed between habitual SOL, self-reported daytime sleepiness, or sleeping pill use and rapid eGFR decline. Our findings regarding SOL align with previous cohort studies,^38^^,^^39^ suggesting that variability in sleep latency may not consistently translate into long-term kidney function decline.

Regarding sleeping pill use, our findings differ from a large cohort study in Taiwan,^40^ where an association with kidney function decline was reported. However, that study did not adjust for other sleep characteristics, which may confound the relationship. In addition, sleeping pill use is highly heterogeneous, because individuals take these medications for various reasons, dosages, and durations. Some may use them intermittently rather than chronically, which could diminish their cumulative impact on renal function. Furthermore, the underlying sleep disturbances leading to medication use may vary in severity, making it difficult to establish a direct relationship with eGFR decline.

For daytime sleepiness, our findings contrast with studies from the UK and France, which reported an association with kidney function decline.^39^^,^^41^ In the French study, a less sensitive eGFR decline cutoff classified one-third of participants as having rapid renal decline, potentially influencing the results. In the UK study, the population attributable risk was only 1.1%, making sleepiness the least significant factor identified. Given that daytime sleepiness is heavily influenced by lifestyle, stress, and work schedules, rather than biological sleep disturbances alone, its role in kidney function decline may be neither strong nor consistent.

Given the limited and inconsistent literature on these associations, further research using objective sleep assessments and longitudinal follow-ups is needed to better understand the relationship between SOL, sleeping pill use, daytime sleepiness, and kidney function decline.

### Strengths and Limitations

This cohort study has several strengths that enhance the validity and reliability of its findings. First, the comprehensive data collection at baseline allowed for well-adjusted statistical models and minimized potential biases. Furthermore, the stratified analysis enabled the exploration of relationships across different age groups, and the use of nap duration to assess daytime napping, rather than a simple binary variable, facilitated the detection of a dose-response relationship, adding depth to the findings. In addition, the study’s examination of both absolute annual eGFR decline and percent change in eGFR provided a comprehensive assessment of kidney function over time, strengthening the study's conclusions. Furthermore, because most previous studies relied on secondary data analysis, researchers often prioritize reporting only significant results, contributing to publication bias, because studies with nonsignificant findings are less likely to be published. This selective reporting can distort the overall understanding of the relationship between sleep and kidney function. However, our study examines multiple sleep characteristics and presents a comprehensive analysis, regardless of statistical significance. This approach provides a more balanced and transparent perspective.

Notwithstanding these strengths, our study is subject to certain limitations that warrant acknowledgment. One key limitation is the limited generalizability of the findings. The study population is from a specific region in Iran, which may not be representative of other populations. In addition, reliance on self-reported sleep data, without the use of validated tools such as the Pittsburgh Sleep Quality Index, limits the accuracy and comprehensiveness of sleep assessments. The lack of objective measurements such as actigraphy or polysomnography could introduce reporting bias because subjective sleep assessments may not accurately reflect actual sleep patterns.

## Conclusion

We observed a strong protective effect of regular daytime napping on kidney function in adults aged ≥ 50 yrs, with evidence of a dose-response relationship. In addition, sleeping < 7 h was linked to a higher risk of rapid renal function decline in younger individuals. Addressing sleep habits may be crucial in strategies aimed at reducing kidney function decline and CKD. Further studies are needed to explore these factors and their relationship with age more thoroughly.

## Disclosure

All the authors declared no competing interests.

## Data Availability Statement

The datasets analyzed during the current study are among the datasets of FACS which can be accessed through direct connection with FACS authorities.

## Author Contributions

MA and JR conceptualized and designed the study. SAM, HMV, AD, and HA contributed to data acquisition. MA and HMV conducted the statistical analysis. MA, SAM, KF, and ZR interpreted the findings and drafted the manuscript. All the authors reviewed and approved the final version of the manuscript.

## Supplementary Material

Supplementary File (PDF) **Table S1.** Baseline characteristics of the study population based on sleep duration and daytime napping. **Table S2.** Baseline characteristics of the study population, based on subjective sleep onset latency, use of sleeping pills, and daytime sleepiness. **Table S3.** Crude and adjusted incidence relative risk of models which developed for the relationship between sleep profile variables and rapid eGFR decline in all participants. STROBE checklist.

## Footnotes

Supplementary File (PDF)
**Table S1.** Baseline characteristics of the study population based on sleep duration and daytime napping. **Table S2.** Baseline characteristics of the study population, based on subjective sleep onset latency, use of sleeping pills, and daytime sleepiness. **Table S3.** Crude and adjusted incidence relative risk of models which developed for the relationship between sleep profile variables and rapid eGFR decline in all participants. STROBE checklist.

## References

[1] Bikbov B., Purcell C.A., Levey A.S., Smith M., Abdoli A., Abebe M. (2020). Global, regional, and national burden of chronic kidney disease, 1990–2017: a systematic analysis for the Global Burden of Disease Study 2017. Lancet.

[2] Jadoul M., Aoun M., Masimango I.M. (2024). The major global burden of chronic kidney disease. Lancet Glob Health.

[3] Berns J.S. (2014). Routine screening for CKD should be done in asymptomatic adults … selectively. Clin J Am Soc Nephrol.

[4] Ramar K., Malhotra R.K., Carden K.A. (2021). Sleep is essential to health: an American Academy of Sleep Medicine position statement. J Clin Sleep Med.

[5] Reutrakul S., Van Cauter E. (2018). Sleep influences on obesity, insulin resistance, and risk of type 2 diabetes. Metabolism.

[6] Krittanawong C., Tunhasiriwet A., Wang Z. (2019). Association between short and long sleep durations and cardiovascular outcomes: a systematic review and meta-analysis. Eur Heart J Acute Cardiovasc Care.

[7] Greenlund I.M., Carter J.R. (2022). Sympathetic neural responses to sleep disorders and insufficiencies. Am J Physiol Heart Circ Physiol.

[8] Wynchank D., Bijlenga D., Penninx B.W. (2019). Delayed sleep-onset and biological age: late sleep-onset is associated with shorter telomere length. Sleep.

[9] Jia M., Li M. (2024). Association of cardiometabolic index with sleep quality in adults: a population-based study. Sci Rep.

[10] Geng T., Li X., Ma H., Heianza Y., Qi L. (2022). Adherence to a healthy sleep pattern and risk of chronic kidney disease: the UK Biobank study. Mayo Clin Proc.

[11] Li Q., Shan Y., Liao J. (2024). Association of daytime napping with incidence of chronic kidney disease and end-stage kidney disease: a prospective observational study. PLoS One.

[12] Xu S., Jin J., Dong Q. (2023). Association between sleep duration and quality with rapid kidney function decline and development of chronic kidney diseases in adults with normal kidney function: the China health and retirement longitudinal study. Front Public Health.

[13] Sasaki S., Yoshioka E., Saijo Y., Kita T., Tamakoshi A., Kishi R. (2014). Short sleep duration increases the risk of chronic kidney disease in shift workers. J Occup Environ Med.

[14] Bo Y., Yeoh E-k, Guo C. (2019). Sleep and the risk of chronic kidney disease: a cohort study. J Clin Sleep Med.

[15] Cheungpasitporn W., Thongprayoon C., Gonzalez-Suarez M.L. (2017). The effects of short sleep duration on proteinuria and chronic kidney disease: a systematic review and meta-analysis. Nephrol Dial Transplant.

[16] Homayounfar R., Farjam M., Bahramali E. (2023). Cohort Profile: the Fasa Adults Cohort Study (FACS): a prospective study of non-communicable diseases risks. Int J Epidemiol.

[17] Siddiquee A.T., Lee S.K., Kim S., Lee M.-H., Kim H.J., Shin C. (2023). All-cause and major-cause mortality associated with sleep latency in the Korean Genome and Epidemiology Study (KoGES): a population-based prospective cohort study. Lancet Healthy Longev.

[18] Delgado C., Baweja M., Crews D.C. (2022). A Unifying Approach for GFR Estimation: Recommendations of the NKF-ASN Task Force on Reassessing the Inclusion of Race in Diagnosing Kidney Disease. Am J Kidney Dis.

[19] Stevens L.A., Schmid C.H., Greene T. (2010). Comparative performance of the CKD Epidemiology Collaboration (CKD-EPI) and the Modification of Diet in Renal Disease (MDRD) Study equations for estimating GFR levels above 60 mL/min/1.73 m^2^. Am J Kidney Dis.

[20] Levey A.S., Coresh J., Greene T. (2007). Expressing the Modification of Diet in Renal Disease Study equation for estimating glomerular filtration rate with standardized serum creatinine values. Clin Chem.

[21] Nelson R.G., Grams M.E., Ballew S.H. (2019). Development of risk prediction equations for incident chronic kidney disease. JAMA.

[22] Poustchi H., Eghtesad S., Kamangar F. (2018). Prospective epidemiological research studies in Iran (the Persian Cohort Study): rationale, objectives, and design. Am J Epidemiol.

[23] Jetté M., Sidney K., Blümchen G. (1990). Metabolic equivalents (METS) in exercise testing, exercise prescription, and evaluation of functional capacity. Clin Cardiol.

[24] Ye Y., Zhang L., Yan W. (2019). Self-reported sleep duration and daytime napping are associated with renal hyperfiltration and microalbuminuria in an apparently healthy Chinese population. PLoS One.

[25] Sun H., Qin K., Zou C. (2021). The association of nighttime sleep duration and quality with chronic kidney disease in middle-aged and older Chinese: a cohort study. Sleep Med.

[26] Yamamoto R., Nagasawa Y., Iwatani H. (2012). Self-reported sleep duration and prediction of proteinuria: a retrospective cohort study. Am J Kidney Dis.

[27] McMullan C.J., Curhan G.C., Forman J.P. (2016). Association of short sleep duration and rapid decline in renal function. Kidney Int.

[28] Hering D., Esler M.D., Schlaich M.P. (2013). Chronic kidney disease: role of sympathetic nervous system activation and potential benefits of renal denervation. EuroIntervention.

[29] Lin H.Y.-H., Hung C.-C., Chang Y.-H. (2015). Nonapnea sleep disorders in patients younger than 65 years are significantly associated with CKD: a nationwide population-based study. PLoS One.

[30] Weil B.R., Mestek M.L., Westby C.M. (2010). Short sleep duration is associated with enhanced endothelin-1 vasoconstrictor tone. Can J Physiol Pharmacol.

[31] Vgontzas A.N., Zoumakis E., Bixler E.O. (2004). Adverse effects of modest sleep restriction on sleepiness, performance, and inflammatory cytokines. J Clin Endocrinol Metab.

[32] Dijk D.-J., Groeger J.A., Stanley N., Deacon S. (2010). Age-related reduction in daytime sleep propensity and nocturnal slow wave sleep. Sleep.

[33] Feinsilver S.H. (2021). Normal and abnormal sleep in the elderly. Clin Geriatr Med.

[34] Duffy J.F., Willson H.J., Wang W., Czeisler C.A. (2009). Healthy older adults better tolerate sleep deprivation than young adults. J Am Geriatr Soc.

[35] Wang Y., Jiang G., Hou N. (2023). Effects and differences of sleep duration on the risk of new-onset chronic disease conditions in middle-aged and elderly populations. Eur J Intern Med.

[36] Jiang B., Tang D., Dai N. (2023). Association of self-reported nighttime sleep duration with chronic kidney disease: China Health and Retirement Longitudinal Study. Am J Nephrol.

[37] Faraut B., Nakib S., Drogou C. (2015). Napping reverses the salivary interleukin-6 and urinary norepinephrine changes induced by sleep restriction. J Clin Endocrinol Metab.

[38] Sasaki S., Yoshioka E., Saijo Y. (2018). A prospective cohort study of insomnia and chronic kidney disease in Japanese workers. Sleep Breath.

[39] Jaussent I., Cristol J.-P., Stengel B. (2016). Impact of sleep disturbances on kidney function decline in the elderly. Eur Respir J.

[40] Liao C.-Y., Chung C.-H., Lu K.-C. (2021). Taking sleeping pills and the risk of chronic kidney disease: a nationwide population-based retrospective cohort study. Front Pharmacol.

[41] Zhang H., Wang B., Chen C. (2022). Sleep patterns, genetic susceptibility, and incident chronic kidney disease: a prospective study of 370 671 participants. Front Neurosci.

