# Supplementary material for: Effect of Sleep Characteristics on Rapid Estimated GFR Decline in Adults with Normal Kidney Function
Source: Kidney Int Rep. 2025 Apr 3;10(6):1733–41. doi: 10.1016/j.ekir.2025.03.042 (PMC12232969; doi:10.1016/j.ekir.2025.03.042)
Supplement: Supplementary File (PDF) — Table S1. Baseline characteristics of the study population based on sleep duration and daytime napping. Table S2. Baseline characteristics of the study population, based on subjective sleep onset latency, use of sleeping pills, and daytime sleepiness. Table S3. Crude and adjusted incidence relative risk of models which developed for the relationship between sleep profile variables and rapid eGFR decline in all participants. STROBE checklist. [file mmc1.pdf]

Table S1. Baseline characteristics of the study population based on sleep duration and daytime napping. To assess the association between variables, we used the chi-squared test for categorical variables and ANOVA test for continuous variables.

		Sleep Duration				Daytime Napping		
**Variables**	**Overall**	**<7 h**	**7-9 h**	**>=9 h**	**P-value**	**No**	**Yes**	**P-value**
Age (year)	49.8 ±8.4	52 ±8.1	49.5 ±8.2	48.3 ±8.5	**<0.001**	50.7 ±8.4	49.2 ±8.3	**<0.001**
eGFR (ml/min/1.73m^2)	88.5 ±10.6	87.1 ±10.5	88.9 ±10.6	89.1 ±10.6	**<0.001**	89.1 ±10.7	88.1 ±10.5	**0.0133**
BMI (kg/m2)	25.9 ±4.7	25.8 ±4.5	25.9 ±4.7	25.9 ±4.7	0.800	25.2 ±4.5	26.3 ±4.7	**<0.001**
Female	1510 (54)	376 (52)	690 (53)	444 (57)	0.083	593 (53)	917 (54)	0.351
Socioeconomic status					0.672			**<0.001**
Low	937 (33)	231 (32)	435 (33)	271 (35)		445 (40)	492 (29)	
Moderate	937 (33)	247 (34)	428 (33)	262 (34)		359 (32)	578 (34)	
High	936 (33)	247 (34)	444 (34)	245 (31)		322 (29)	614 (36)	
Fars ethnicity	1496 (53)	407 (56)	725 (55)	364 (47)	**<0.001**	656 (58)	840 (50)	**<0.001**
Education years					0.596			**<0.001**
Low	1372 (49)	369 (51)	630 (48)	373 (48)		614 (55)	758 (45)	
Moderate	989 (35)	237 (33)	469 (36)	283 (36)		362 (32)	627 (37)	
High	449 (16)	119 (16)	208 (16)	122 (16)		150 (13)	299 (18)	
Currently Smoker	473 (17)	112 (15)	221 (17)	140 (18)	0.417	219 (19)	254 (15)	**0.002**
Uncontrolled HTN	453 (16)	109 (15)	220 (17)	124 (16)	0.565	176 (16)	277 (16)	0.563
Diabetes	369 (13)	116 (16)	160 (12)	93 (12)	**0.029**	151 (13)	218 (13)	0.714
CVD	306 (11)	111 (15)	123 (9)	72 (9)	**<0.001**	142 (13)	164 (10)	**0.016**
NSAID use	137 (5)	48 (7)	64 (5)	25 (3)	**0.01**	65 (6)	72 (4)	0.074
ACEi/ARB use	192 (7)	60 (8)	85 (7)	47 (6)	0.194	73 (6)	119 (7)	0.534
Physical Activity					**<0.001**			**<0.001**
Low	942 (34)	158 (22)	419 (32)	365 (47)		261 (23)	681 (40)	
Moderate	936 (33)	288 (40)	441 (34)	207 (27)		398 (35)	538 (32)	
High	932 (33)	279 (38)	447 (34)	206 (26)		467 (41)	465 (28)	
Dyslipidemia	845 (30)	215 (30)	396 (30)	234 (30)	0.955	340 (30)	505 (30)	0.915
Daytime Napping	1684 (60)	266 (37)	770 (59)	648 (83)	**<0.001**			
Sleep duration								**<0.001**
<7 h	725 (26)					459 (41)	266 (16)	
7-9 h	1307 (47)					537 (48)	770 (46)	
>=9 h	778 (28)					130 (12)	648 (38)	
Prolonged sleep latency	189 (7)	127 (18)	41 (3)	21 (3)	**<0.001**	100 (9)	89 (6)	**<0.001**
Sleeping Pills Use	200 (7)	62 (9)	82 (6)	56 (7)	0.16	88 (8)	112 (7)	0.239
Daytime sleepiness	899 (32)	215 (30)	409 (31)	275 (35)	**0.046**	339 (30)	560 (33)	0.08

Variables are presented as mean ±standard deviation or numbers (percentages). Abbreviations: eGFR, estimated glomerular filtration rate; BMI, body mass index; HTN, hypertension; CVD, cardiovascular disease; NSAID, nonsteroidal anti-inflammatory drug; ACEi, angiotensin-converting enzyme inhibitor; ARB, angiotensin II receptor blocker.

Table S2. Baseline characteristics of the study population based on subjective sleep onset latency, use of sleeping pill, and daytime sleepiness. To assess the association between variables, we used the chi-squared test for categorical variables and ANOVA test for continuous variables.

	Subjective SOL			Sleeping Pills Use			Daytime sleepiness		
**Variables**	**<=60min**	**>60min**	**P-value**	**No**	**Yes**	**P-value**	**No**	**Yes**	**P-value**
Age (year)	49.6 ±8.4	51.9 ±8	**<0.001**	49.8 ±8.4	49.9 ±7.8	0.891	49.1 ±8.4	51.3 ±8.1	**<0.001**
eGFR (ml/min/1.73m2)	88.5 ±10.6	85.9 ±9.7	**<0.001**	88.7 ±10.6	86.8 ±9.9	**0.018**	88.7 ±10.6	88.1 ±10.6	0.143
BMI (kg/m2)	25.9 ±4.7	25.7 ±4.7	0.515	25.8 ±4.7	26 ±4.3	0.704	25.9 ±4.7	25.7 ±4.6	0.239
Female	1342 (53)	138 (73)	**<0.001**	1385 (53)	125 (62)	**0.010**	1013 (53)	497 (55)	0.259
Socioeconomic status			0.801			0.515			**0.044**
Low	846 (34)	66 (35)		875 (34)	62 (31)		615 (32)	322 (36)	
Moderate	840 (33)	59 (31)		863 (33)	74 (37)		632 (33)	305 (34)	
High	837 (33)	64 (34)		872 (33)	64 (32)		664 (35)	272 (30)	
Fars ethnicity	1388 (55)	95 (50)	0.396	1394 (53)	102 (51)	0.510	1081 (57)	415 (46)	<0.001
Education years			**<0.001**			0.731			**<0.001**
Low	1208 (48)	119 (63)		1272 (49)	100 (50)		864 (45)	508 (57)	
Moderate	897 (36)	58 (31)		917 (35)	72 (36)		694 (36)	295 (33)	
High	418 (17)	12 (6)		421 (16)	28 (14)		353 (18)	96 (11)	
Currently Smoker	423 (17)	24 (13)	0.116	441 (17)	32 (16)	0.744	346 (18)	127 (14)	**0.009**
Uncontrolled HTN	393 (16)	31 (16)	0.913	414 (16)	39 (20)	0.178	290 (15)	163 (18)	**0.047**
Diabetes	323 (13)	30 (16)	0.249	334 (13)	35 (18)	0.054	228 (12)	141 (16)	**0.006**
CVD	265 (11)	31 (16)	**0.012**	259 (10)	47 (24)	**<0.001**	191 (10)	115 (13)	**0.027**
NSAID use	121 (5)	14 (7)	0.096	118 (5)	19 (10)	**0.002**	86 (5)	51 (6)	0.171
ACEi/ARB use	162 (6)	25 (13)	**<0.001**	164 (6)	28 (14)	**<0.001**	109 (6)	83 (9)	**<0.001**
Physical Activity			0.08			0.078			0.052
Low	850 (34)	56 (30)		866 (33)	76 (38)		633 (33)	309 (34)	
Moderate	840 (33)	77 (41)		864 (33)	72 (36)		664 (35)	272 (30)	
High	833 (33)	56 (30)		880 (34)	52 (26)		614 (32)	318 (35)	
Dyslipidemia	748 (30)	69 (37)	**0.046**	787 (30)	58 (29)	0.729	590 (31)	255 (28)	0.173
Daytime Napping	1524 (60)	89 (47)	**<0.001**	1572 (60)	112 (56)	0.239	1124 (59)	560 (62)	0.080
Sleep duration			**<0.001**			0.160			**0.046**
<7 h	595 (24)	127 (67)		663 (25)	62 (31)		510 (27)	215 (24)	
7-9 h	1224 (49)	41 (22)		1225 (47)	82 (41)		898 (47)	409 (45)	
>=9 h	704 (28)	21 (11)		722 (28)	56 (28)		503 (26)	275 (31)	
Prolonged sleep latency				164 (7)	25 (13)	**0.001**	125 (7)	64 (8)	0.568
Sleeping Pills Use	170 (7)	25 (13)	**0.001**				117 (6)	83 (9)	**0.003**
Daytime sleepiness	785 (31)	64 (34)	0.568	816 (31)	83 (42)	**0.003**			

Variables are presented as mean ±standard deviation or numbers (percentages). Abbreviations: SOL, sleep onset latency; eGFR, estimated glomerular filtration rate; BMI, body mass index; HTN, hypertension; CVD, cardiovascular disease; NSAID, nonsteroidal anti-inflammatory drug; ACEi, angiotensin-converting enzyme inhibitor; ARB, angiotensin II receptor blocker.

Table S3. Crude and adjusted incidence relative risk of models which developed for the relationship between sleep profile variables and rapid eGFR decline in all participants

	Crude Model		Adjusted Model*	
Sleep profile variables	RR (95% CI)	P-value	RR (95% CI)	**P-value**
Daily sleep duration (reference: 7-9 hours)				
less than 7 hours	1.12 (0.90-1.39)	0.328	1.08 (0.85-1.36)	0.528
more than 9 hours	1.08 (0.87-1.34)	0.498	1.23 (0.97-1.55)	0.090
Daytime napping	**0.79 (0.66-0.95)**	**0.013**	**0.81 (0.66-1.00)**	**0.047**
Prolonged sleep onset latency	1.26 (0.91-1.75)	0.171	1.22 (0.87-1.73)	0.252
Sleeping pill use	0.87 (0.60-1.28)	0.488	0.78 (0.52-1.18)	0.240
Daytime Sleepiness	0.95 (0.78-1.16)	0.616	0.89 (0.72-1.10)	0.288

* Adjusted models include all sleep variables and is adjusted for age, gender, ethnicity [Fars, not fars], baseline eGFR, uncontrolled HTN, diabetes mellitus, history of cardiovascular disease [yes, no], BMI, socioeconomic status [tetiles], education level [categorical], physical activity [tertiles], smoker [yes,no], dyslipidemia [yes, no] , and using NSAID or ACEi/ARB.

STROBE Statement—Checklist of items that should be included in reports of ***cohort studies***

	Item No	Recommendation	Page No
**Title and abstract**	1	(*a*) Indicate the study's design with a commonly used term in the title or the abstract<br><br>(*b*) Provide in the abstract an informative and balanced summary of what was done and what was found	1
**Introduction**			
Background/rationale	2	Explain the scientific background and rationale for the investigation being reported	3
Objectives	3	State specific objectives, including any prespecified hypotheses	3
**Methods**			
Study design	4	Present key elements of study design early in the paper	4
Setting	5	Describe the setting, locations, and relevant dates, including periods of recruitment, exposure, follow-up, and data collection	4
Participants	6	(*a*) Give the eligibility criteria, and the sources and methods of selection of participants. Describe methods of follow-up<br><br>(*b*) For matched studies, give matching criteria and number of exposed and unexposed	4
Variables	7	Clearly define all outcomes, exposures, predictors, potential confounders, and effect modifiers. Give diagnostic criteria, if applicable	5
Data sources/ measurement	8*	For each variable of interest, give sources of data and details of methods of assessment (measurement). Describe comparability of assessment methods if there is more than one group	5
Bias	9	Describe any efforts to address potential sources of bias	6,7
Study size	10	Explain how the study size was arrived at	4
Quantitative variables	11	Explain how quantitative variables were handled in the analyses. If applicable, describe which groupings were chosen and why	7
Statistical methods	12	(*a*) Describe all statistical methods, including those used to control for confounding<br><br>(*b*) Describe any methods used to examine subgroups and interactions<br><br>(*c*) Explain how missing data were addressed<br><br>(*d*) If applicable, explain how loss to follow-up was addressed<br><br>(*e*) Describe any sensitivity analyses	7

**Results**			
Participants	13*	(a) Report numbers of individuals at each stage of study—eg numbers potentially eligible, examined for eligibility, confirmed eligible, included in the study, completing follow-up, and analysed	
		(b) Give reasons for non-participation at each stage	7,8
		(c) Consider use of a flow diagram	
Descriptive data	14*	(a) Give characteristics of study participants (eg demographic, clinical, social) and information on exposures and potential confounders	
		(b) Indicate number of participants with missing data for each variable of interest	7,8 table1
		(c) Summarise follow-up time (eg, average and total amount)	
Outcome data	15*	Report numbers of outcome events or summary measures over time	7

Main results	16	(*a*) Give unadjusted estimates and, if applicable, confounder-adjusted estimates and their precision (eg, 95% confidence interval). Make clear which confounders were adjusted for and why they were included	8 Table 2 & 3
		(*b*) Report category boundaries when continuous variables were categorized	
		(*c*) If relevant, consider translating estimates of relative risk into absolute risk for a meaningful time period	
Other analyses	17	Report other analyses done—eg analyses of subgroups and interactions, and sensitivity analyses	Table 2
**Discussion**			
Key results	18	Summarise key results with reference to study objectives	8
Limitations	19	Discuss limitations of the study, taking into account sources of potential bias or imprecision. Discuss both direction and magnitude of any potential bias	12
Interpretation	20	Give a cautious overall interpretation of results considering objectives, limitations, multiplicity of analyses, results from similar studies, and other relevant evidence	9-11
Generalisability	21	Discuss the generalisability (external validity) of the study results	9-11
**Other information**			
Funding	22	Give the source of funding and the role of the funders for the present study and, if applicable, for the original study on which the present article is based	13

*Give information separately for exposed and unexposed groups.

Note: An Explanation and Elaboration article discusses each checklist item and gives methodological background and published examples of transparent reporting. The STROBE checklist is best used in conjunction with this article (freely available on the Web sites of PLoS Medicine at http://www.plosmedicine.org/, Annals of Internal Medicine at http://www.annals.org/, and Epidemiology at http://www.epidem.com/). Information on the STROBE Initiative is available at http://www.strobe-statement.org.
